# Acid Yellow 9 Azo Dye Gets the Blues: An Optical Spectroscopy and DFT Study of Unusual Photochemistry in Multilayer Films with PAH and Chitosan

**DOI:** 10.3390/molecules30193850

**Published:** 2025-09-23

**Authors:** Mikhail Kim, Tristan H. Borchers, Monica Lin, Christopher J. Barrett

**Affiliations:** 1 Department of Chemistry, McGill University, Montreal, QC H3A 0B8, Canada; 2 School of Chemistry, University of Birmingham, Edgbaston, Birmingham B15 2TT, UK

**Keywords:** azo dyes, photochemistry, chitosan, DFT, multilayered films, polyelectrolytes

## Abstract

Multilayer and free-standing films self-assembled from water-soluble anionic azo dye acid yellow 9 (AY9) and both poly(allylamine hydrochloride) (PAH) and chitosan (CS) cationic polyelectrolytes were fabricated from water solution using a layer-by-layer (LbL) technique and characterized by UV–Vis and Raman spectroscopy. Observations were made of a strong, unexpected, and highly unusual colour change from deep red to a distinct dark blue upon exposure of the multilayer films to an acidic environment. The colour change was attributed to the multilayer films only and was not observed either for the polymer or the dye alone, or their mixture in water solution, nor when cast as free-standing films. The significant shift to blue colour of the absorption peaks was quantified with UV–Vis spectroscopy, and a proposed explanation is presented based on density functional theory (DFT) calculations exploring possible and most likely acid-base equilibria configurations of the azo dye that result from being self-assembled.

## 1. Introduction

**Figure 1:**
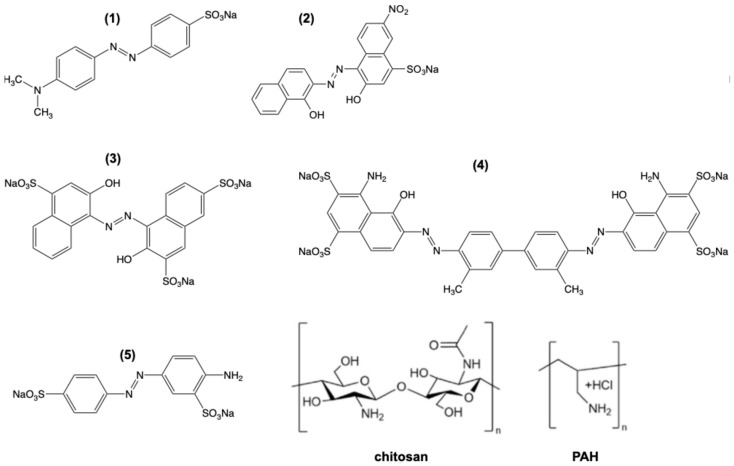
Molecular structures of polymers and azo dyes discussed in this study (**1**) methyl orange, (**2**) eriochrome black T, (**3**) hydroxynaphthol blue, (**4**) evans blue, and (**5**) acid yellow 9.

The large class of azobenzene dye derivatives has long been used as food and clothing dyes since the 1830s [1] due to the wide variety of colours obtainable, depending on the substituents on the benzene rings and depending on the conditions such as pH, temperature, pressure, the presence of metal ions, or upon *trans-cis* isomerization induced by light irradiation [2]. The strong and condition-dependent colour changes of some azo dyes have been used for decades as acid-base indicators (e.g., well-known methyl orange (MO, Figure 1(1)) and methyl red) and complexometric titration (e.g., azo-dyes calconcarboxylic acid, fast sulphone black F, eriochrome black T, Figure 1(2)). The vast majority of these azo dyes are most commonly red, orange, or yellow in colour. Rarely, azo dyes can also display an intrinsic blue colour (evans blue, hydroxynaphthol blue) when incorporated synthetically into a complex molecular structure of a conjugated multi-rings separated by azo groups (Figure 1(3,4)). An azo-dye AY9 (Figure 1(5)), also known as fast yellow AB (or formerly as food dye E105, now forbidden in the USA and Europe) studied here, is well-known to change its colour from yellow to orange upon an acid protonation in water solutions.

In our previous research [3,4] we developed stable (water insoluble) multilayer films made using a layer-by-layer assembly technique of water-soluble cationic polyelectrolytes and water-soluble anionic azo food dyes with the ability to reversibly re-solubilize upon exposure to visible light. The mechanism for this reversible solubility changes was proposed to be the *trans-cis* isomerization of azo dye crosslinkers within multilayer films leading to changes in the molecular geometry and, as the result, changes in interaction between azo-dyes and the polymer matrix. Our goal was to fabricate ‘green materials’, from low toxicity biocompatible polymers, azo food dyes, and water as the solvent, avoiding use of organic solvents, and concentrated acids or bases. It is well-known that polar solvents such as water facilitate the back *cis-trans* isomerization of azo-dyes, in a strong and rate-enhancing solvato-chromic effect [5,6]. Moreover, the azo-dyes used in our study were targeted to be FDA-approved azo food-grade dyes containing methoxy- or/and hydroxy- groups in ortho-position relatively to the azo-group, that have been demonstrated to have a much faster thermal *cis-trans* back isomerization, greatly assisting and enhancing some applications, but prohibiting direct spectroscopic analysis of the extremely short-lived *cis* form [7]. As the result, it was difficult to track the interconversion between the geometric and differently coloured isomers or observe the colour of the *cis* isomers directly upon light radiation by visually recording the *cis* isomers’ spectra distinct from the *trans* isomer colour [8]. The relatively simple molecular structure of AY9 containing only one azo group and no naphthalene rings would historically be expected to yield a yellow colour, far unlike the blue colours observed for complex multi-azo and multi-ring azo dyes such as eriochrome black T, evans blue, and hydroxynaphtol blue, mentioned above. However, it was a surprise for our group to observe a sudden, stable, and sharp colour change from red to deep blue in a multilayer films PAH-AY9 at a low pH.

Literature research did not result in uncovering any previous mention or explanation of this highly anomalous and surprising colour change to blue of AY9 observed in our laboratories. Various colour changes of azo dyes due to *trans-cis* isomerization have been well studied and described [8,9], but such prior observations and explanations were eliminated from consideration as the reason for this strong ‘blueing’ of AY9 in our multilayers, now outlined otherwise in the following sections. It was reported previously that azo dyes might have polymorphs with different properties, including different colours, for example methyl yellow dye in its orthorhombic form is yellow, and in monoclinic form as an orange colour [10]. Crystal structures of AY9 as sodium and magnesium salts, as well as structures of barium and calcium salts of two analogues, were studied [11], but despite having different crystal structure and physical properties, the salts’ colour changed less significantly from yellow to just orange and red (both AY9 salts exhibit a dark red colour), and no deeper into the green or blue regions of the visible spectrum. Alkyl derivatives of Sudan III were synthesized [12] with alkyl chain lengths varied from 4 to 18, where the colour did change for different crystal structures, but only from orange to red. The crystallization within a multilayer film is known to be possible [13,14,15] since confinement effects of closely packed layers and the interfacial interactions can stabilize specific crystal structures. However, this strong change we observed to a deep blue colour occurred only in acidic water solutions. Our experiments showed instead that, at very low pH (0.44), AY9 still was soluble and did not precipitate, hence it is questionable that the dye formed solid crystal structures in the well-hydrated and disordered multilayer films, unless the interaction with polyelectrolytes led to a decrease in the solubility.

Two examples from previous research in the science of dye colour shed some light and provided a possible approach to study the phenomenon. One study [16] investigated the underlying chemistry of blue-black to pink-orange colour changes in cooked lobster. This colour change is attributed to thermal denaturation of the astaxanthin–α-crustacyanin protein complex, with consequent releasing of a neutral α-hydroxyketone form of astaxanthin, with further possibility of formation of its enolate form observed with UV–Vis absorption maxima shift up to 160 nm. The results of this UV–Vis spectral analysis and quantum chemical calculations of a model system that retained the trimethylated α-hydroxycyclohexenone of astaxanthin but replaces the rest of the molecule with a more simple α-β-unsaturated ketone at the 6-position of the ring demonstrated the capacity of the quantum calculation methods to reproduce experimental spectra well, and confirms that enolization of astaxanthin within the protein environment causes the significant spectral shift. The astaxanthin–protein complex in the lobster carapace is a natural example of a strongly interacting polymer–dye host–guest system that resembles the self-assembled multilayer films of our study. In studies of animal photoreceptors [17], it was demonstrated that the pigment retinal in its complex with protein opsin (rhodopsin) has light absorption peaks from 366 nm in birds [18], to 420–560 nm in humans [19], to 690 nm in some fungi [20]. Such dramatic absorption peak shifts for the same pigment were linked to the opsin primary structure and its configuration within the rhodopsin complex17 resulting in different protonation states, hydrophobicity of the environment, and strength of hydrogen bonds between the protein and the pigment.

Previously, our group demonstrated [21] that in an aqueous environment within a multilayer film poly(acrylic acid) (PAA) and PAH acid-base equilibria can decrease substantially upon self-assembly for PAA and increase significantly in the case of PAH. These pKa shifts were as large as 4 pH units and depended on the number of polymer layers in the films. Such shifting of apparent pKa of an azo dye within multilayer film could lead to protonated states of the dye now accessible at a low (but ‘safe’) pH that cannot otherwise be observed that would result in shifting of absorption spectra of the dye, but only at extremely low pH values that would lead to chemical decomposition. To investigate and attempt to rationalize this phenomenon of AY9 colour change with computational chemistry, density functional theory (DFT) and time-dependent DFT (TD-DFT) was used for modelling of the various exited states to predict theoretical UV–Vis spectra of the polymer–azo–dye system in different acid-base conditions. Previous studies demonstrated good predictability for the exited states of azobenzene derivatives utilizing TD-DFT [22,23,24,25,26,27]. In our study presented here, we compare modelled excited states for AY9 in different protonation forms, in different solvent environments with different dielectric constant, simulating the host–guest polymer matrix assemblies.

## 2. Results

### 2.1. Multilayer Film Characterization

Multilayer films of PAH-AY9 and CS-AY9 were observed to be uniform after fabrication and drying, red in colour, and insoluble in water under a wide range of pH, salt, and temperature conditions (in the dark, or ambient laboratory illumination). However, upon exposure to 460 nm LED light under running water nearing sunlight intensities, the films became water soluble and disassembled as demonstrated in Figure S2A (insert). The portion of the films covered with a mask remained intact. As seen in Figure S2A, there appear to be two events occurring throughout the first 20 scans of the experiment: a peak shifting of the band at 350 nm (Figure S2B), and the disappearance of the band at 486 nm (Figure S2C). The peak shifting is an indication of isomerization from *trans* azobenzene to *cis* azobenzene [8,28,29]. Additionally, very little absorbance loss is seen in the first 20 scans when analyzing the band at 365 nm, in significant contrast to that seen at 486 nm. After the complete disappearance of the band at 486 nm, the remaining bands begin to lose prominence and eventually no absorbance is seen.

Another well-known interesting property of azo dye thin polymer films is the ability to inscribe microscale diffraction volume gratings on the films’ surface using 532 nm polarized light (Figure S3). The photo-fabrication of the substantial grooves and peaks on the surface has long been attributed as resulting from the unique *trans-cis* isomerization of azo dyes within the polymer matrix, as this effect is only observed in azo systems that isomerize, and not with any other colouration by non-isomerizing dye systems [2]. Thus, in the absence of direct observation of the ultra-fast isomerization in the AY9 system, the successful photo-inscription of diffraction gratings served as an indirect implication of the ability of AY9 to isomerize, if too quickly to track spectroscopically by available laser laboratory equipment.

To study the unexpected and surprising pH-driven colour change of the films in detail that was observed serendipitously, multilayer samples were submerged into water solution of various dilutions of hydrochloric acid, where a pH-dependent colour change was observed immediately. The films were gently dried in a jet of air and then analyzed immediately via UV–Vis spectroscopy. The blue colour of the films observable at the lowest pH conditions did not disappear upon a gentle heating with hot air at 40 °C for 10 min. From this observation at elevated temperature, we can deduce that the blue colour cannot be associated with the formation of any unstable *cis* isomer. The colour change was completely reversible, with the red–blue colour transition demonstrated reproducibly and indistinguishably over many repeated cycles of pH immersion back and forth from low to neutral (Figure S4).

**Figure 2:**
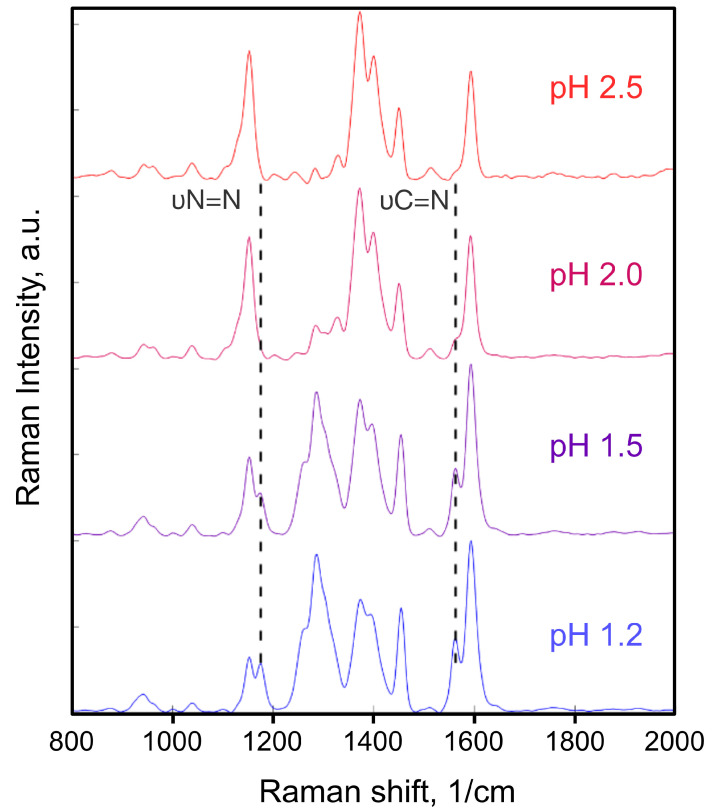
Raman bands of PAH/AY9 a multilayer film equilibrated in solutions of different pH.

The protonation of the azo bond was determined through Raman spectroscopy, using a confocal Raman microscope. The multilayer film was dipped into four acidic solutions from pH 2.5 to pH 1.2, dried, and then monitored with the 532 nm probe laser. As seen in Figure 2, the vibration bands of C=N at 1563 cm^−1^ and the stretching bands of protonated N=N at 1176 cm^−1^ and 1420 cm^−1^ increased as pH decreased, while the band of the stretching vibration of C−N at 1153 cm^−1^ reduced in prominence with respect to the remainder of the spectrum [30,31]. These changes in the Raman spectra indicate changes in the electronic structure caused by protonation of the azo bond.

### 2.2. UV–Vis Spectra of AY9 Solutions and Multilayer Films

**Figure 3:**
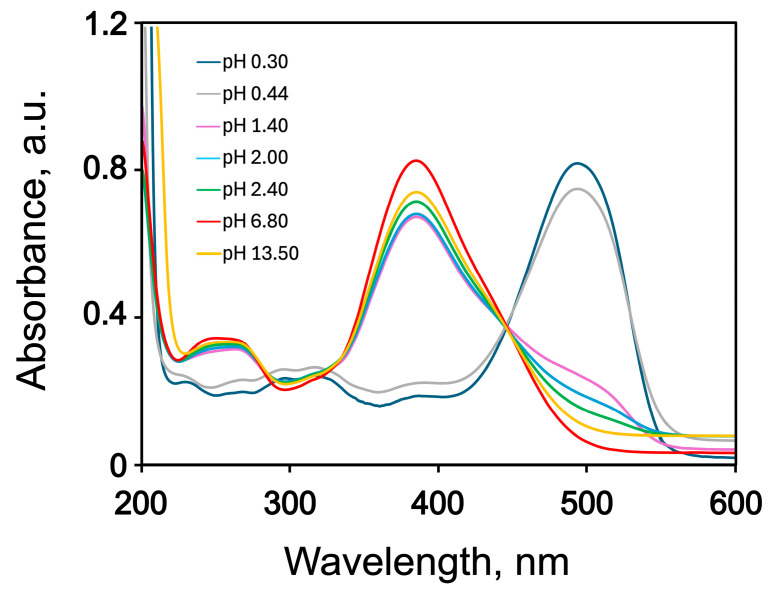
Experimental UV–Vis spectra of 0.1 mM AY9 water solutions at different pH values. Nonzero absorbances near 600 nm are not implied optical properties of the dyes, but assumed artifacts.

AY9 as a typical azo dye changes its colour upon protonation, and like many others can be used as an acid-base indicator. With AY9, the colour changes from yellow to orange, and eventually to red with decreasing pH. The colour of water solutions of AY9 even at pH as low as 0.24, however, remained red, at the low pH colour extremity. The experimental UV–Vis spectra of water solutions of AY9 are presented in Figure 3. For comparison, spectra of two azo dyes having distinct blue colour at pH = 7 are shown in Figure S5. The spectra of AY9 in water solutions display broad peaks due to hydrogen bonds formed between the dye and water. Non-aqueous solutions (Figure S6) exhibit more distinct absorption peaks in the UV region, and narrower peaks at 420 nm, similar in shape to the absorption peaks at 390 nm of water solutions at high pH.

Water solutions of AY9 display spectra typical for azobenzenes with a broad and distinct peak in the visible region attributed to an n-π* electron transition of the azo group, and a smaller peak in the UV region, attributed to a π-π* electron transition of azo-group. Exact assignment of these absorption peaks will be discussed in the DFT calculation section. From Figure 3, one can see the clear appearance of a 500 nm absorption peak with decreasing pH, and an almost total disappearance of a 390 nm peak at pH values between 1.40 and 0.44. At the same time, the peak from UV region at 263 nm for basic solutions red-shifted to 307 nm for the acidic solutions. The transition from yellow to orange to red was observed upon titration of AY9 in water solution with 1 M solution of hydrochloric acid for the pH range from 1.80 to 0.60, respectively.

**Figure 4:**
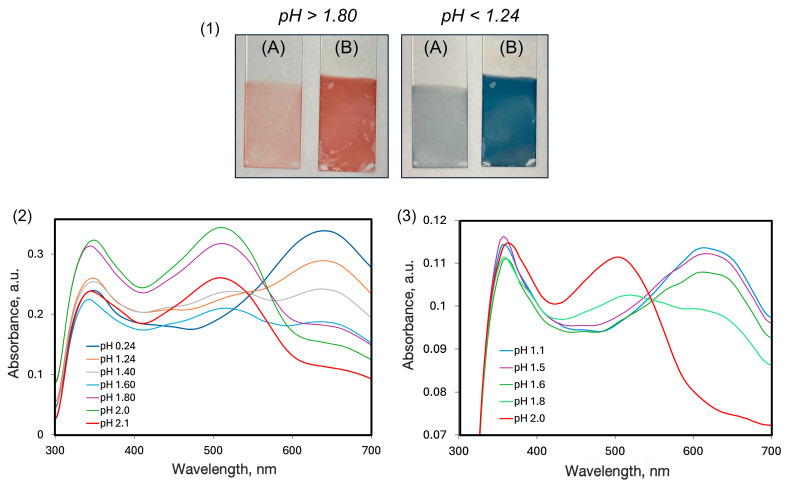
(**1**) Change in colour of 90 BL films PAH/AY9 at different pH values, self-assembled in either the presence of NaCl (**A**) or in pure distilled water (**B**). UV–Vis spectra of multilayer films at different pH values, for (**2**) PAH/AY9 and (**3**) CS/AY9, assembled in distilled water.

UV–Vis spectra of the multilayer films PAH/AY9 and CS/AY9 are presented in Figure 4, together with photographs of multilayers deposited on the glass substrates at different pH values. For multilayer films of the same thickness of 90 BL assembled in the presence of NaCl, which commonly aids in assembling thick and robust multilayer films, the colour of the layers is less pronounced (UV–Vis spectra of the multilayer films are shown in Figure S7). This might be explained by a less compact layer deposition in the presence of strong electrolytes. Noticeably, the pH threshold of 1.1 for the colour change to blue is the same in both cases. For the system CS/AY9 (Figure 4(3)) the colour transition occurs at pH = 1.8. In all UV–Vis spectra, the absorption peak of AY9 at 500 nm was observed for the red-coloured films, that then disappeared as the films changed to blue with this new peak appearing at 650 nm. Remarkably, the absorption peak in the UV region observed in multilayer films at 350 nm was sharper and red shifted, compared to the spectra of the pure dye in water. Also, this UV peak did not shift upon decreasing pH. Due to the high absorption of the glass substrates at wavelengths below 300 nm, the entire multilayer film spectra cannot be compared with spectra of AY9 in water solutions.

In contrast to the AY9/PAH systems, multilayer films of the same thickness, composed from PAH and MO, dis not exhibit any such dramatic colour change to blue at low pH (Figure S8). Also, we did not observe any colour change to blue at low pH for any of the many azo dyes studied previously [3,4], in otherwise identical multilayer films self-assembled with the same polycation PAH and various aqueous food azo dyes (allura red, amaranth, sunset yellow, or tartrazine), nor in any of those multilayers composed with polyanion polyacrylic acid and aqueous cationic azo dyes bismark brown Y, or bismarck brown R. Thus, efforts to rationalize this curious red-to-blue effect focused on the structural characteristics unique to the AY9 dye, not present in any of the other azo dyes, namely the ring SO_3_ group in the *meta*- position to the azo bond.

### 2.3. DFT and TD-DFT Calculations

We established the optimized geometry of *trans* and *cis* isomers of AY9 by exploring the energetics over the rotational pathway of the CNNC dihedral angle (Figure S9). The geometry of only the *trans* isomer with dihedral angle CNNC = 180° was used to further calculations as the starting geometry input, since we did not expect the *cis* isomer to be present in multilayer films at such low pH, because the back *cis-trans* isomerization is catalyzed strongly by acids [5]. Moreover, if the blue colour was attributed to the *cis* isomer, it would have disappeared upon heating to accelerate the *cis-trans* thermal isomerization, in contrast to observations of a stable blue colour after formation. The difference in energy between *trans* and *cis* isomers for all three functionals used was found to be in the same tight range (Table S1), from 0.70 to 0.73 eV, that agreed well with earlier reported values for a rotation mechanism of 0.87 eV in one paper [32], and 0.74 eV in another [33].

**Figure 5:**
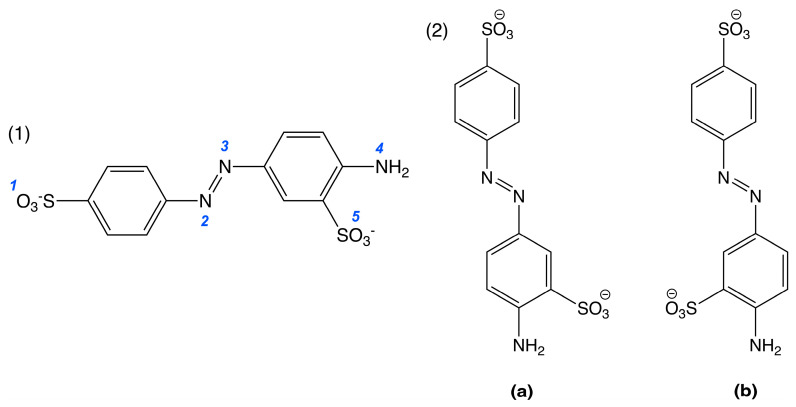
*Trans* isomers of AY9: (**1**) The blue numbers indicate the possible proton-accepting sites, and (**2**) the two possible geometric ring-azo bond conformers (**a**) and (**b**) of AY9.

Optimized geometries and energies were then calculated for AY9 for the most probable sites being protonated, that we determined were most likely from initial assessment. AY9 has five potential proton-accepting sites (Figure 5(1)). For azo dye acid-base indicators, it was previously determined that the azo group becomes protonated first, giving the indicator’s distinct colour change [22,34]. In our study, we compared energies of the AY9 molecule with different degrees of protonation, compared to the energy of fully deprotonated AY9 (Table 1). Additionally, we considered two conformers of AY9 for our calculations, with different benzene rings conformations about the azo bond with respect to the SO_3_ ring substituent (Figure 5(2)). The results for the absolute energy values are given in Table S1 and for relative energies in Table 1.

**Figure 6:**
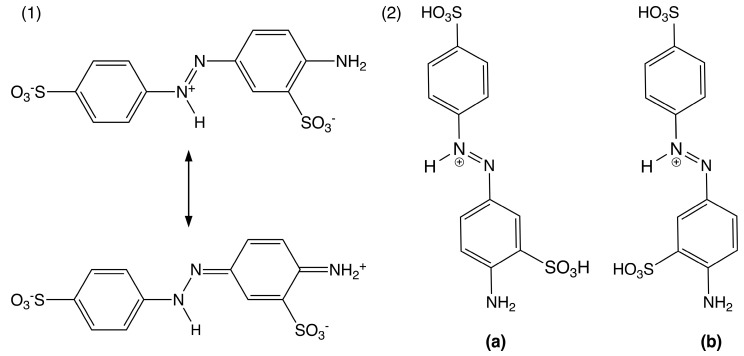
(**1**) Resonance structures of protonated AY9 (charge of −1). (**2**) Protonated structures of (**a**) and (**b**) conformers for protonated AY9 (charge of +1).

These calculations demonstrated a decrease in the energy with each protonation step. This decrease can be explained as a stabilizing of the protonated NN bond of AY9 by resonance on the first protonation step (Figure 6(1)) and an increase in entropy on each protonation step due to release of water molecules into the bulk, previously structured around the charged sulfo groups. The energy difference between conformers (**a**) and (**b**) was not found to be significant for most of the protonated states, except the state with a total charge of +1, where the conformer (**b**) demonstrated a higher energy, possibly due to steric effects of the sulfo-group and interaction with the azo-group hydrogen atom (Figure 6(2)).

Based on optimized ground-state geometries, absorption maxima were then obtained as vertical excitation energies calculated using time-dependent DFT (TD-DFT) with calculations for 20 singlet-singlet excited states, with the three functionals B3LYP, CAM-B3LYP, and LC-ωHPBE which have been designed in part to offer a better description of excited states than standard functionals [23,35,36]. The long-range corrected CAM-B3LYP functional was employed, as it was reported to provide a more accurate description of excited states, especially for charge transfer states, than B3LYP [22]. The TD-DFT calculation output provides the excitation energies and oscillator strengths. The energies were then converted to wavelength in nm, using the formula *E* = *hc/λ* (where *c* = 3 × 10^8^ m/s the speed of light, and *h* = 6.63 × 10^−34^ J⋅Hz^−1^, the Planck constant). The oscillator strength is dimensionless and relates to the probability of absorption or emission upon transitions between molecular energy levels [37,38].

The calculations show (Table 2) that the most intense (the highest oscillator strength *f*) absorption peaks for fully deprotonated states are associated with the electronic transition S_2_ for B3LYP and LC-ωHPBE functionals, while for CAM-B3LYP the most intense peaks are linked to the S_7_ excitation state. The accepted experimental value of approximately 3.22 eV (absorption peak at pH = 13.5, Figure 3) compares the closest with a value of 3.04 eV predicted with the B3LYP functional. Upon protonation, using B3LYP, the electronic transition S_1_ becomes more stable after protonation of three sites. For the CAM-B3LYP and LC-ωHPBE functionals, the electronic transition S_1_ becomes more stable than S_2_ after the first protonation of the azo bond. Comparison of (**a**) and (**b**) AY9 conformers for different degrees of protonation is provided in Figure S3.

**Figure 7:**
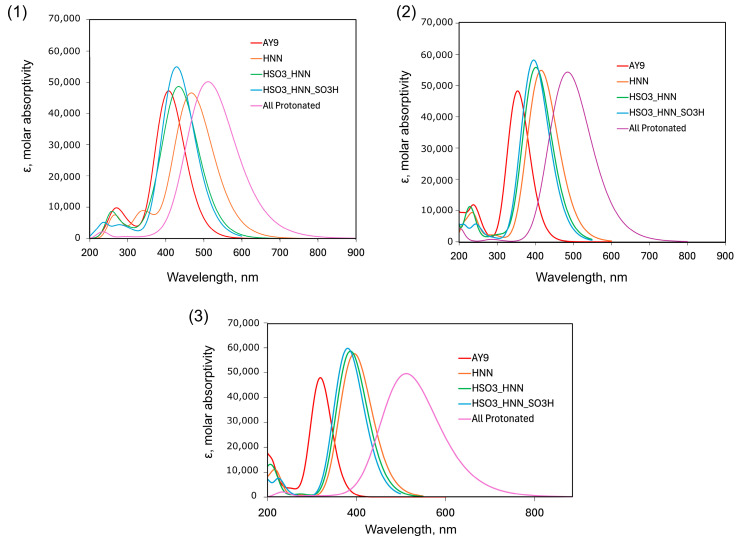
Result of TD-DFT calculations of AY9 absorption spectra with different sites of protonation using the basis set 6-31g(p,d) and a CPCM (water) model, with functionals: (**1**) B3LYP, (**2**) CAM-B3LYP, and (**3**) LC-ωHPBE, in molar absorptivity with units of L⋅mol^−1^⋅cm^−1^.

Theoretical adsorption spectra predicted with Gaussian software from the TD-DFT calculations of the excited states are plotted in Figure 7 for five different protonation states. For all functionals, one can notice red-shifting of the absorption peak of AY9 upon the first protonation step of the azo group. In the subsequent two protonation steps, a slight blue-shifting of the spectra was observed. The model spectra for the all-protonated state predicted the longest wavelength of the absorption peak up to 510 nm, attributed again to a large spectral red shift compared to all previously protonated states. Although a general trend of significant red-shifting arises from the calculations, the full extent of the red shift to 650 nm observed experimentally in the multilayer films was not predicted, perhaps due to an incomplete model of strong solvent effects present.

It was found that a particular excited state can be an admixture of several transitions between different molecular orbitals. For example, the excited state 2 is an admixture of two transitions from MO #89 to MO #93, and from MOs #92 to #93, hence an exact description and analysis of this state should be considered complex and non-trivial. Calculation of NTOs, on another hand, can simplify the description. By transforming the standard MO representations into a more compact form, NTOs express each excited state, when possible, as a single dominant pair of orbitals: the transition occurring from an occupied NTO (so-called ‘particle’) to the unoccupied NTO (‘hole’) [39]. NTO generation was completed for the two functionals B3LYP and CAM-B3LYP, for excited states S_1_ and S_2_. The results are given in Figure S10, where the shape of the MO allows one to assign the n-π* and π-π* electron transitions of the calculated excited states. For example, for the functional B3LYP calculation, for fully deprotonated AY9 (Figure S10A), the S_1_ transition (2.66 eV energy gap, 466 nm absorption peak) has zero probability to occur (*f* = 0.0000), corresponding to the n-π* transition, while the S_2_ transition (3.04 eV energy gap, 408 nm absorption peak) has a much higher probability (*f* = 1.1516) and expected to be associated with the π-π* transition. For fully protonated AY9, S_1_ and S_2_ un-occupied NTOs appeared to lose electron localization on the NN bond. Instead, S_1_ and S_2_ transitions correspond to bonding and non-bonding NTOs of the benzene rings to anti-bonding NTOs delocalized across multiple atomic centres of the benzene rings and the azo group. The transition for S_1_ (2.03 eV energy gap, 611 nm absorption peak) had a smaller probability (*f* = 0.0072) corresponding to the n-π* transition, while the S_2_ transition (2.36 eV energy gap, 525nm absorption peak) with a higher occurrence probability (*f* = 0.2236) is linked to the π-π* transition.

### 2.4. Effect of the Counter-Polymer

To estimate effect of the polymer matrix on the UV–Vis absorption shift, we calculated the shift values between multilayer film spectra and pure AY9 in water solutions, such as ‘protonated’ at pH = 0.50, and ‘deprotonated’ at pH = 7.00 (Table 3). The type of the polymer used in the multilayer films was not found to significantly affect the value of the shift of either the protonated or deprotonated states. This is also in agreement with experimentally observed values of pH of the red-to-blue colour change, that were not significantly different between two counter-polycations PAH (at pH = 1.1) and CS (at pH = 1.8). It could well be that the red-shifting effect was a result mainly of the proximity to the polymer amino groups and did not depend significantly on the more-distant backbone structure of the polymer.

Additionally, based on some previous reports [40,41], we hypothesize that the polymer matrix with a lower dielectric constant than water [42,43,44,45] might red-shift the UV–Vis spectra of AY9. Hence, we performed TD-DFT calculations with a CPCM model using heptane (*ε* = 1.9) [46] to mimic the aliphatic PAH environment (*ε* = 2–3) [44,45]. We had observed a red shift of wavelength in the simulated spectra for fully protonated AY9 (Figure S11) for two functionals, with the highest shift of 100 nm observed from the B3LYP functional, and the smallest shift of 10 nm for the LC-ωHPBE functional. Such simulation of the polymer environment using a solvent environment with a more similar dialectic constant to the polymer host could give a better estimation on how the polymer matrix effects excited states of the azo dye with minimum computational cost. However, for a more correct assessment, it is necessary to use explicit polymer molecules for the TD-DFT calculations.

## 3. Discussion

The all-protonated state was considered as purely hypothetical in water solution, since the pKa values of similar sulfo-containing azo-dyes compounds are very low [47]. However, it was previously demonstrated that in multilayered films PAH/PAA the pKa values of the weak acid-base groups were shifted significantly [21,48]. Hence, it is possible that all protonated states could potentially be achieved in multilayer films with PAH and CS, considering these apparent pKa deviations. Thus, three functionals were compared for calculation of excited states of the AY9 molecule. An attempt to explain red-shifting of absorption peaks in UV–Vis spectra was made via a hypothesis that the dye becomes fully protonated in the multilayer film environment. Such an unusually fully protonated state might be expected due to a high proximity of positively charged amino groups of the polymers to the dye’s proton-accepting sites in multilayer film, and by the non-equilibrium local environment with a low dielectric constant of the polymer matrix. However, the phenomena of unusual colour change of AY9 within polymer matrix of PAH or CS cannot be explained only by full protonation of the dye at low pH. Contrary to the hypothesis of the fully protonated AY9, each protonation step should make the AY9 molecule more positively charged, making it more difficult to add a subsequent proton. If such effect is prevalent, further analysis and reasoning would need to be pursued.

For possible future studies, a more complex TD-DFT exploration project could be undertaken, with the polymer and water molecules explicitly included in the calculations. Including large polymer molecules would be prohibitively computationally costly and complicated, so a more oligomeric (5 to 10 polymer unit) environment could be pursued to be used instead. To test the hypothesis of a pKa change influencing acid-base equilibria and colour, DFT calculations [49] could be performed for AY9 alone and within the polymer matrix and compared to the experimental data (initial experimental determination of pKa of AY9 is shown in Figure S12). As was discussed in the introduction, multilayer films are capable of creating a unique and highly localized and non-equilibrium electromagnetic environment, and future DFT packages may well be able to capture these effects in a computationally accessible manner in the future. Other future work on modeling these curious colour-change systems may well also benefit from consideration of other processes that are possible, for example those reported for styryls and their hetero-derivatives such as photoinduced cyclization [50], or cyclobutane formation [51]. Such other transformations have been known to significantly affect the absorption properties in mixtures, and these effects could be explored either to reasonably rule them out, or to be taken into account.

In addition to posing a curious and anomalous scientific puzzle, this red-shifting of an azo dye within multilayer films to deep blue may well find some applications in acid-base titration and sensor design. The phenomena of shifting pKa equilibria in multilayer assemblies might be used to study azo dyes or small molecules at unusually high protonation states using visible light and at an accessible pH. Also, infrared (IR) light-induced *trans-cis* isomerization of azo-dyes was proposed to be used in drug delivery [52]. Usually, for an azo-dye to have absorption of high wavelength light, an additional substitute must be included in the benzene ring, such as fluorine substitutes, that requires a complex synthetic pathway or using complex systems [53,54]. Using this red-shift phenomenon, described in our study, however, one could design a multilayered system of a polymer and an azo-dye with tailorable absorption of azo-dyes in the IR region at physiological pH levels. Such medical applications might also benefit from the fact that PAH and chitosan are biocompatible, and, in case of chitosan, biodegradable, and that the azo dye could in principle be chosen from a group of FDA-approved food-grade low toxicity azo dyes.

## 4. Materials and Methods

### 4.1. LbL Assembly of Thin Films

Acid yellow 9 (4-Amino-1,1′-azobenzene-3,4′-disulfonic acid monosodium salt, dye content 90%), poly(allylamine hydrochloride) (average MW 50,000 g/mol), and chitosan of low molecular weight (50,000–190,000 g/mol), were purchased from Sigma Aldrich (St. Louis, MI, USA), and were used without an additional purification. AY9 was purchased from Sigma Aldrich sold as ‘dye content 90%’ and was re-crystallized from water. Sodium chloride, sodium hydroxide (0.1 M water solution), 37% hydrochloric acid, 98% sulfuric acid, 90% ethanol, dimethyl sulfoxide, and 30% hydrogen peroxide were purchased from ThermoFisher (Waltham, MA, USA) and were used without additional purification. Hydrochloric acid was diluted with distilled water to a 1% by mass concentration.

An automated slide stainer dipping robot Varistain 24–4 (Shandon Scientific Limited, Abbey Ward, UK) was used to sequentially assemble multi-layered films consisting of the polymer crosslinked with the azo dyes. For the substrate, 1′′ × 3′′ microscope glass slides were cleaned by immersing in a ‘piranha solution’ (mixture of sulfuric acid/30% hydrogen peroxide) at room temperature for 24 h. The multilayer deposition process was as follows: the negatively charged treated substrates were immersed in positively charged polymer solutions (0.01 M of mers) for 10 min, followed by three rounds of dipping in separate rinsing baths with deionized (DI) water (pH = 7) for 1 min each. The substrates were then immersed in a 0.01 M solution of the negatively charged AY9 azo dye for 10 min, followed by 3 similar rounds of 1 min rinsing with DI water. This dipping process was repeated to form a film with 90 bilayers (BL) in total. Additionally, 90 BL multilayer films of PAH-AY9 were assembled on the glass substrates otherwise identically, but in the presence of 1% by mass NaCl. A schematic of the dipping process for LbL assembly is shown in Figure S1.

### 4.2. Raman Spectroscopy

Characterization studies were performed on a WITec Alpha 300 R Raman imaging microscope (Witec GmbH, Ulm, Germany) using a 532 nm probe system, at 1 mW power. To achieve optimal signal clarity, spectra were captured with an integration period of 1 s and underwent 25 points of accumulation. Prior to analysis, all spectra were corrected for darkness and intensity using the OSX software package (2018). Analysis was carried out through MATLAB in which the imported raw data would be subject to several data treatment functions including background subtraction using MATLAB function (msbackadj), and the resultant data would then undergo a normalization function, by area under the curve. Following normalization, specific signals of the product and the reagent where selected and integrated; then, the integrated signals would be normalized from 0–1 and plotted against time.

### 4.3. UV–Visible (UV–Vis) Spectroscopy

UV–Vis spectroscopy of the assembled films was performed in transmittance mode using a Varian Cary 300 Biospectrometer from Agilent Technologies (Marshallton, DE, USA), with a clean 2 mm thick glass slide for multilayer measurements, or distilled water in 1 cm quartz cuvettes for solution studies, measured initially as background, and during the measurements as the references. UV–Vis absorption spectra of the dry multilayer films coated on the glass surface were recorded from 300 to 700 nm, in 2 nm increments. UV–Vis absorption spectra of solutions were recorded from 200 nm to 700 nm, in 2 nm increments, and plotted as molar absorptivity vs. wavelength.

### 4.4. pH Measurements

To change pH, 0.1 M water solutions of sodium hydroxide or 1% mass water solutions of hydrochloric acid were used. For the experiments with the multilayer films, solutions with different pH values from 0.2 to 2.2 were prepared. A ThermoScientific Orion Star (Saint Saint Paul, MN, USA) pH-meter was used to measure pH at room temperature 22 °C. The multilayer films on the glass substrates were submerged into 15 mL beakers with solutions of each certain pH for 30 s to equilibrate. The excess of the solution was removed with gentle jet of air, and the film was left to dry for 10 min under fume hood ambient conditions before acquiring UV–Vis spectra.

### 4.5. Photo-Response of the Multilayer Films

Diffraction gratings were written on the fabricated multilayer film surfaces using 532 nm linearly polarized light from a confocal laser, with grating lines inscribed at a spacing distance of 10 μm as described [55]. Reversible solubility of the multilayer films was tested analogously with methods described previously [1,3]. The films deposited on glass substrates were clamped under a slow stream of DI water (pH = 7) with flow rate of 20 mL/s maintained and recycled using an electric pump, at room temperature of approximately 20 °C. The slides were mounted so that half the coated surface was shielded from light behind a black mask, but exposed to the same water flow, ‘upstream’ of the irradiated half of each slide. The black mask allowed for two zones of exposure, with the upper part of the slide being fully masked to prevent any exposure to light (control dark region), while the lower part of the slide was fully exposed to light irradiation. The films were irradiated using LEDs peaking in wavelength at 460 nm to match the azo absorption band, with light powers of 10 mW, placed 15 cm from the surface of the sample. The light-induced disassembly process was paused every 15 min to perform a measurement.

### 4.6. Computational Chemistry

Optimized structures of AY9 were calculated using DFT with functionals B3LYP, LC-ωHPBE, and CAM-B3LYP. Ground-state structures were verified by vibrational analysis and the absence of imaginary frequencies. Bulk solvation effects were accounted for using the CPCM polarizable conductor calculation model [22,23,27]. Geometry optimizations were carried out in environments of water, dimethyl sulfoxide (DMSO), ethanol (EtOH), and heptane (Hep). Basis sets chosen used for all calculations were either 6-31g(d,p) that did not include diffuse functions, or 6-31+g(d,p) that included diffuse functions on second-row atoms. A CPCM model by default was used for non-equilibrium solvation conditions for TD-DFT and for equilibrium conditions for ground state calculations.

Natural Bond Orbital analysis (NBO) was performed for optimized geometries by running the “pop = nbo” command in Gaussian software. Natural Transition Orbitals (NTO) were generated for 3 excited states with the highest oscillator strength from 20 calculated, using the scheme described in the Supporting Information.

### 4.7. Data Processing and Visualization, Computational Chemistry Software

For data processing, visualization, and UV–Vis spectra processing, MATLAB v.23.2.0.2391609 (R2023b), update 2 was used. Molecular structures were prepared using ChemOffice software v.23.1.1.3 and Avogadro v.1.99.0. Computational chemistry calculations were performed using Gaussian 16 software, revision C. 01, running on clusters provided by Cedar and Graham of the Digital Research Alliance of Canada (www.alliancecan.ca, URL accessed on 20 November 2023).

## Figures and Tables

**Table 1:** Relative Gibb’s energy (in Hartree units) at 298.15 K of optimized *trans*-AY9 geometries of different protonated states, with different functionals using the basis set 6-31g(d,p) and a CPCM (water) model. Red font indicates the specific hydrogen atoms added in each case. The benzene ring structures, and double bonds are omitted for clarity.

Protonated State	Total Charge	Functional		
		B3LYP	CAM-B3LYP	LC-ωHPBE
SO_3__NN_NH_2__SO_3_ (a)	−2	0	0	0
SO_3__NN_NH_2__SO_3_ (b)		0.0001	0.0001	0.0004
SO_3__ HNN_NH_2__SO_3_ (a)	−1	−0.4412	−0435	−0.4331
SO_3__HNN_NH_2__SO_3_ (b)		−0.4417	−0.4349	−0.4325
HSO_3__ HNN _NH_2__SO_3_ (a)	0	−0.8743	−0.8648	−0.8636
HSO_3__ HNN _NH_2__SO_3_ (b)		−0.8737	−0.8648	−0.8642
HSO_3__HNN_NH_2__SO_3_H (a)	+1	−1.2935	−1.2817	−1.2825
HSO_3__HNN_NH_2__SO_3_H (b)		−1.2915	−1.2800	−1.2807
HSO_3__HNNH_NH_2_H_SO_3_H (a)	+3	−2.0223	−1.9997	−1.9990
HSO_3__HNNH_NH_2_H_SO_3_H (b)		−2.0229	−2.0010	−1.9995

**Table 2:** Calculated vertical excitation energies in eV and oscillator strength *f* (the values in parentheses) of optimized AY9 geometries for conformer (**a**) for different protonated states, with different functionals using the basis set 6-31g(d,p) and a CPCM (water) model. Red font indicates the hydrogen atoms added. The benzene ring structures and double bonds are omitted for clarity. S_n_ is the excited state of n-order with the highest oscillator strength after S_1_ and S_2_ states.

Protonated State	Excited State	Functional		
		B3LYP	CAM-B3LYP	LC-ωHPBE
SO_3__NN_NH_2__SO_3_	S_1_	2.66 (0.0000)	2.85 (0.0001)	2.92 (0.0001)
	S_2_	3.04 (1.1516)	3.51 (0.0210)	3.89 (1.1832)
	S_n_	4.53 (0.1680)/S8	5.28 (0.1996)/S_7_	7.27 (0.3175)/S_18_
SO_3__HNN_NH_2__SO_3_	S_1_	2.54 (0.0004)	2.98 (1.3539)	3.14 (1.4213)
	S_2_	2.65 (1.1334)	4.09 (0.0242)	4.46 (0.0029)
	S_n_	3.65 (0.1447)/S_8_	5.20 (0.1869)/S_9_	6.95 (0.3695)/S_17_
HSO_3__ HNN _NH_2__SO_3_	S_1_	2.60 (0.0000)	3.09 (1.3784)	3.21 (1.4477)
	S_2_	2.83 (1.1281)	3.98 (0.0009)	4.44 (0.0053)
	S_n_	3.28 (0.1963)/S_3_	6.68 (0.2263)/S_20_	7.14 (0.4329)/S_16_
HSO_3__HNN_NH_2__SO_3_H	S_1_	2.89 (1.3353)	3.13 (1.4363)	3.26 (1.4763)
	S_2_	3.42 (0.0290)	4.32 (0.0224)	4.42 (0.0025)
	S_n_	5.26 (0.0746)/S_14_	6.80 (0.2640)/S_17_	7.20 (0.3345)/S_13_
HSO_3__HNNH_NH_2_H_SO_3_H	S_1_	2.03 (0.0072)	2.43 (0.0408)	2.67 (1.2842)
	S_2_	2.36 (0.2236)	2.56 (1.3002)	2.84 (0.0638)
	S_n_	2.38 (0.5720)/S_3_	6.06 (0.0388)/S_17_	6.98 (0.1485)/S_20_

**Table 3:** Computationally predicted absorption peaks of AY9 in the multilayer films with different polymer matrix conditions, and the absorbance maximum shift in comparison with that determined experimentally in distilled water solution of the dye (nm), based on data from Figure 3 and Figure 4(2,3).

Polymer, Conditions of LbL Fabrication	Deprotonated (Red Colour)		Protonated (Blue Colour)	
	Absorption Peaks	Shift	Absorption Peaks	Shift
PAH, DW	512 348	125 86	642 350	255 88
PAH, NaCl	500 364	113 102	630 358	243 96
CS, DW	505 364	118 102	620 357	233 95

## Supplementary Materials

The following supporting information can be downloaded at: https://www.mdpi.com/article/10.3390/molecules30193850/s1, Figure S1: Scheme of layer-by-layer (LbL) assembly for polycation polymer and anion azo dye cross-linker multilayer films; Figure S2: (A) UV–Vis spectra of PAH/AY9 multilayer films under light-disassembly conditions (460 nm irradiation, 20 mL/s running water). Each trace was acquired 15 min apart; (B) Shifts of absorbance bands over time at 350 nm (shown in (A) in blue box); (C) Shifts in absorbance bands over time at 486 nm (shown in (A) in red box); Figure S3: Diffraction gratings with 10 μm spacing photo-inscribed into the surface of a CS-AY9 LBL thin film with 532 nm linearly polarized light using a confocal laser; Figure S4: Changes in absorbance peaks of AY9 in a multilayer film with PAH upon reversible cycling of pH. Odd numbers correspond to pH 0.2, and even numbers to pH 2.5; Figure S5: Experimental UV–Vis spectra of two blue azo-dyes in water solution at pH 7; Figure S6: Experimental UV–Vis spectra of AY9 in DMSO and ethanol solutions; Figure S7: Experimental UV–Vis spectra of a 90 BL PAH/AY9 multilayer film assembled in the presence of 1 M NaCl, measured at different pH values; Figure S8: Photographs of 90 BL multilayer films of PAH-MO, after being immersed to equilibrium in two contrasting pH solutions; Figure S9: Energy profile calculations for the thermal *cis-trans* back isomerization pathway of AY9 via rotation through the dihedral angle CNNC, predicted by a transition state search through the rotation pathway; Figure S10: Results of NTO analysis of two excited states S_1_ and S_2_ for the AY9 molecule of different protonation sites, using a 6-31g(p,d) basis set, CPCM (implicit), and using functionals: (A) B3LYP or (B) CAM-B3LYP; Figure S11: Theoretical predictions of absorption spectra for AY9 in solvents water and heptane, from TD-DFT calculations with a 6-31g(p,d) basis set, model CPCM (implicit), and functionals: (A) B3LYP, (B) CAM-B3LYP, and (C) LC-ωHPBE of full protonation (sites 1–5); Figure S12: Labels and numbering of atoms in AY9 for DFT calculations; Table S1: Calculated energies of optimized *trans* and *cis* isomers of the fully deprotonated form of AY9, in eV; Table S2: Absolute Gibb’s energy (in Hartree units) at 298.15 K of optimized *trans* AY9 geometries of different protonated states, with different functionals using the basis set 6-31g(d,p) and a CPCM (water) model; Table S3: Calculated vertical excitation energies in eV and oscillator strength f (values in parentheses) optimized for the two (a) and (b) conformers of AY9 for different protonated states, with different functionals using basis set 6-31g(d,p) and a CPCM (water) model.

## Abbreviations

The following abbreviations are used in this manuscript:

PAH	Poly(allylamine hydrochloride)
AY9	Acid Yellow 9
LbL	Layer-by-layer
UV–Vis	Ultraviolet–visible
DFT	Density functional theory
TD-DFT	Time-dependent density functional theory
DW	Distilled water
PAA	Poly(acrylic acid)
