# Supplementary material for: Acid Yellow 9 Azo Dye Gets the Blues: An Optical Spectroscopy and DFT Study of Unusual Photochemistry in Multilayer Films with PAH and Chitosan
Source: Molecules. 2025 Sep 23;30(19):3850. doi: 10.3390/molecules30193850 (PMC12525761; doi:10.3390/molecules30193850)
Supplement: Supplementary file 1 [file molecules-30-03850-s001.zip › molecules-3854204-supplementary.pdf]

Supplementary Information for:

Acid Yellow 9 Azo Dye Gets the Blues: An Optical Spectroscopy and DFT Study of Unusual Photochemistry in Multilayer Films with PAH and Chitosan

Mikhail Kim[1], Tristan H. Borchers[1,2], Monica Lin[1], Christopher J. Barrett[1,*]

[1] McGill University, Montreal, QC, Canada
[2] University of Birmingham, Birmingham, United Kingdom
* Correspondence: christopher.barrett@mcgill.ca

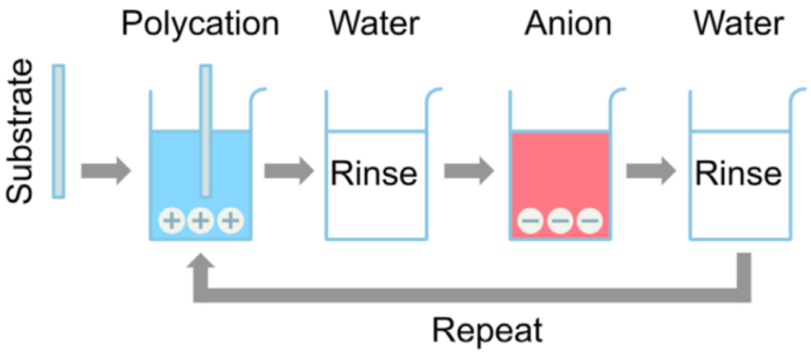

Figure S1 Scheme of layer-by-layer (LbL) assembly for polycation polymer and anion azo dye cross-linker multilayer films.

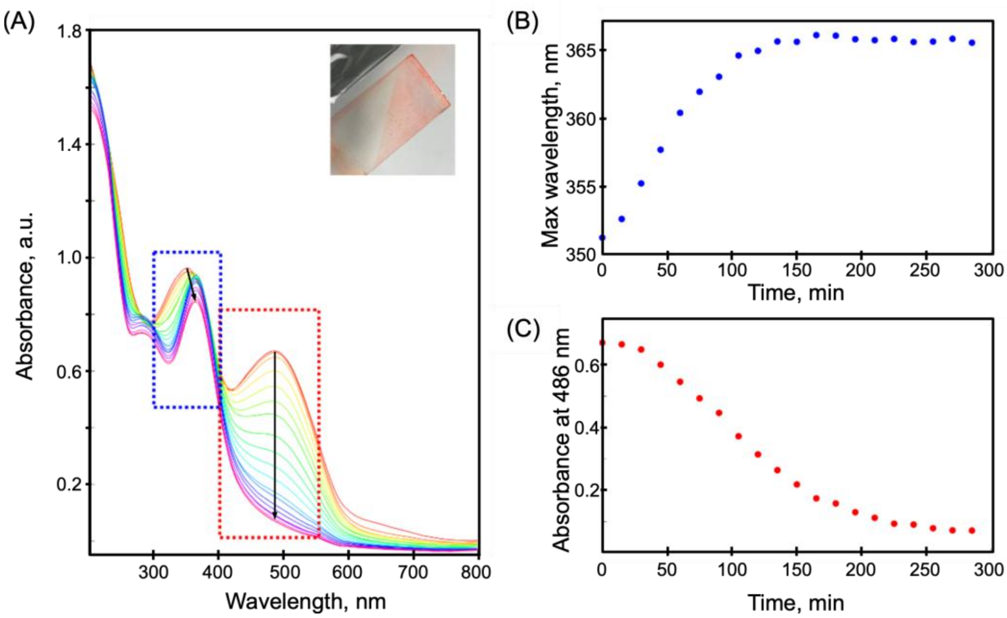

Figure S2 (A) UV-Vis spectra of PAH/AY9 multilayer films under light-disassembly conditions (460 nm irradiation, 20 mL/s running water). Each trace was acquired 15 mins apart; (B) Shifts of Absorbance bands over time at 350 nm (shown in (A) in blue box); (C) Shifts in Absorbance bands over time at 486 nm (shown in (A) in red box).

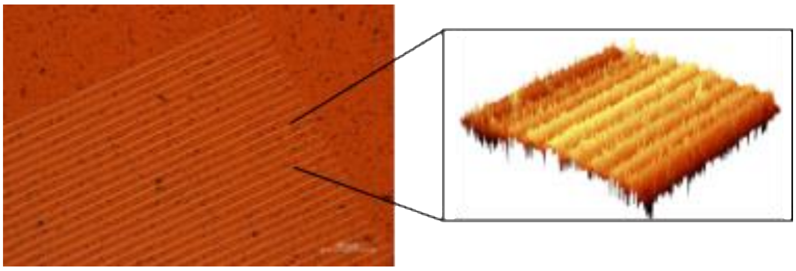

Figure S3 Diffraction gratings with 10μm spacing photo-inscribed into the surface of a CS-AY9 LBL thin film with 532 nm linearly polarized light using a confocal laser.

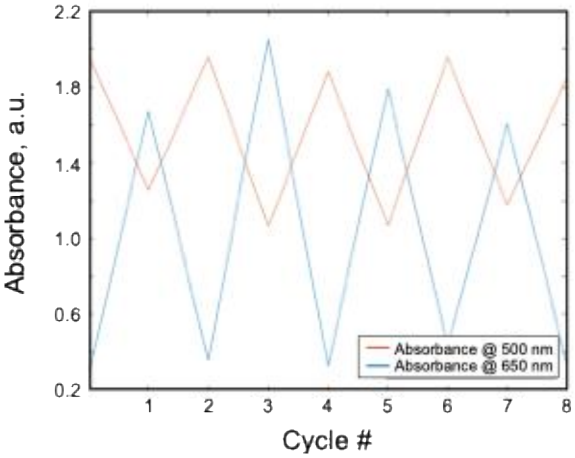

Figure S4 Changes in absorbance peaks of AY9 in a multilayer film with PAH upon reversible cycling of pH. Odd numbers correspond to pH 0.2, and even numbers to pH 2.5.

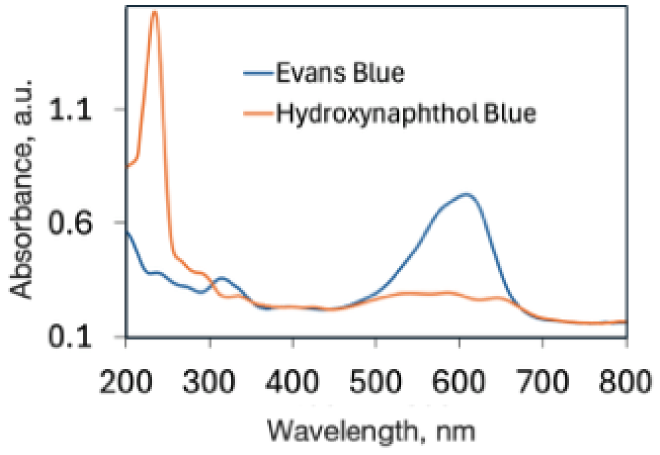

Figure S5 Experimental UV-Vis spectra of two blue azo-dyes in water solution at pH 7.

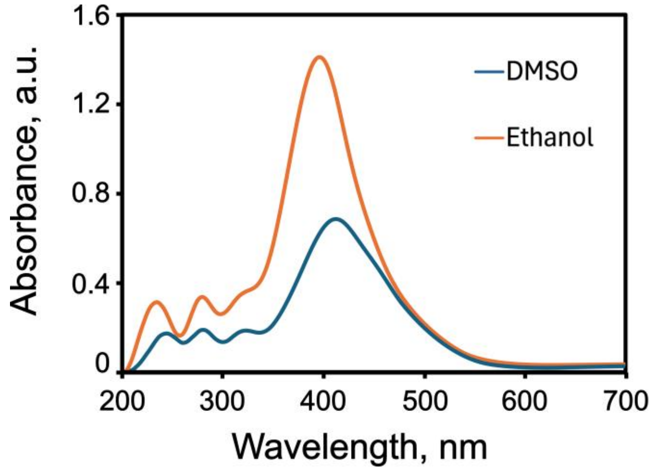

Figure S6 Experimental UV-Vis spectra of AY9 in DMSO and Ethanol solutions.

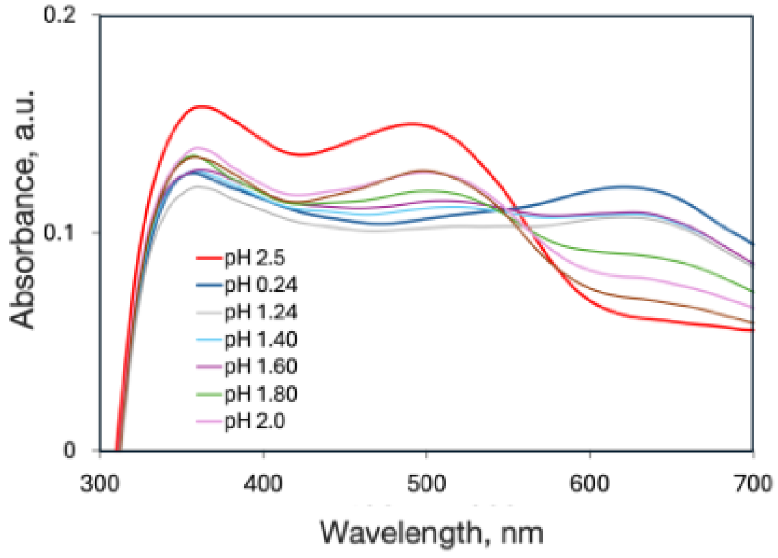

Figure S7 Experimental UV-Vis spectra of a 90 BL PAH/AY9 multilayer film assembled in the presence of 1M NaCl, measured at different pH values.

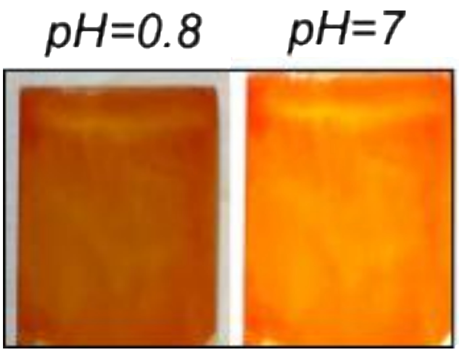

Figure S8 Photographs of 90 BL multilayer films of PAH-MO, after being immersed to equilibrium in two contrasting pH solutions.

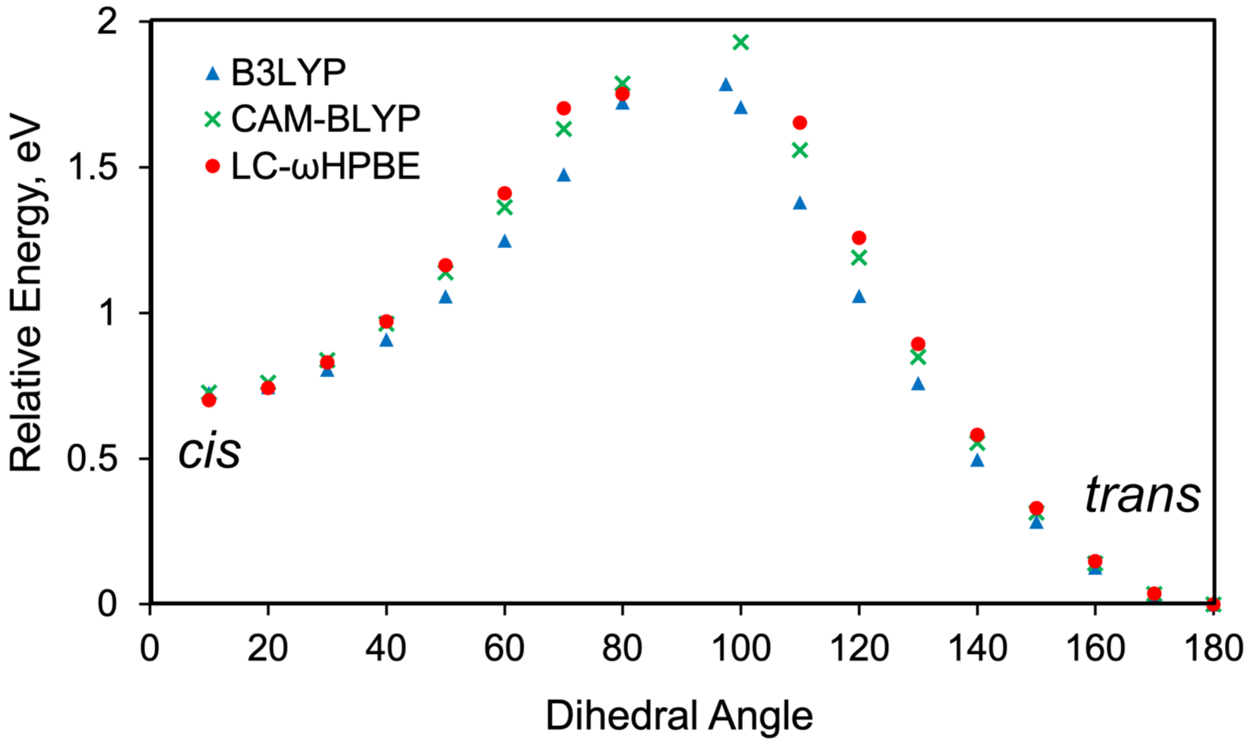

Figure S9 Energy profile calculations for the thermal *cis-trans* back isomerization pathway of AY9 *via* rotation through the dihedral angle CNNC, predicted by a transition state search through the rotation pathway. This search was conducted by full geometry optimization under constraint of the CNNC dihedral angle, which was incremented in steps of 10°. The optimization was performed with the functionals described and a 6-31g(d,p) basic set to establish the geometry of minimum energy states for *trans* and *cis* isomers, confirmed to be in minima as without any imaginary vibrational frequencies.

Table S1 Calculated energies of optimized *trans* and *cis* isomers of the fully deprotonated form of AY9, in eV. Calculations used the 6-31g(d, p) basis set, with functionals as indicated.

**Functional**	***trans* isomer 180°**	***cis* isomer 10°**	$E_{trans\text{-}cis}$, *eV*
B3LYP	−51011.494	−51010.768	0.73
CAM-B3LYP	−50998.576	−50997.848	0.73
LC-ωHPBE	−50985.077	−50984.375	0.70

Table S2 Absolute Gibb's energy (in Hartree units) at 298.15 K of optimized *trans* AY9 geometries of different protonated states, with different functionals using the basis set 6-31g(d,p) and a CPCM (water) model. Red font indicates the hydrogen atoms added; benzene ring structures, and double bonds are omitted for the clarity.

Protonated state	Total charge	Functional		
		B3LYP	CAM-B3LYP	LC-ωHPBE
SO_3_NN_NH_2_SO_3 (a)	−2	−1874.7066	−1874.2293	−1873.7329
SO_3_NN_NH_2_SO_3 (b)		−1874.7065	−1874.2292	−1873.7325
SO_3_HNN_NH_2_SO_3 (a)	−1	−1875.1478	−1874.6643	−1874.1660
SO_3_HNN_NH_2_SO_3 (b)		−1875.1483	−1874.6642	−1874.1654
HSO_3_ HNN _NH_2_SO_3 (a)	0	−1875.5809	−1875.0941	−1874.5965
HSO_3_ HNN _NH_2_SO_3 (b)		−1875.5803	−1875.0941	−1874.5971
HSO_3_HNN_NH_2_SO_3H (a)	+1	−1876.0001	−1875.5110	−1875.0154
HSO_3_HNN_NH_2_SO_3H (b)		−1875.9981	−1875.5093	−1875.0136
HSO_3_HNNH_NH_2H_SO_3H (a)	+3	−1876.7289	−1876.2290	−1875.7319
HSO_3_HNNH_NH_2H_SO_3H (b)		−1876.7295	−1876.2303	−1875.7324

Table S3 Calculated vertical excitation energies in eV and oscillator strength *f* (values in parentheses) optimized for the two (**a**) and (**b**) conformers of AY9 for different protonated states, with different functionals using basis set 6-31g(d,p) and a CPCM (water) model. Red font denotes the hydrogen atoms added; benzene ring structures, and double bonds are omitted for the clarity.

**Protonated state**	**Excited state**	**Functional**		
		**B3LYP**	**CAM-B3LYP**	**LC-ωHPBE**
SO_3_NN_NH_2_SO_3 (a)	S_1	2.66 (0.0000)	2.85 (0.0001)	2.92 (0.0001)
	S_2	3.04 (1.1516)	3.51 (0.0210)	3.89 (1.1832)
	S_n	4.53 (0.1680)/S8	5.28 (0.1996)/S_7	7.27 (0.3175)/S_{18}
SO_3_NN_NH_2_SO_3 (b)	S_1	2.64 (0.0004)	2.84 (0.0003)	2.91 (0.0011)
	S_2	3.03 (1.1475)	3.50 (1.1756)	3.89 (1.1737)
	S_n	4.53 (0.1166)/S_8	6.56 (0.2495)/S_{20}	6.80 (0.3225)/S_{13}
SO_3_HNN_NH_2_SO_3 (a)	S_1	2.54 (0.0004)	2.98 (1.3539)	3.14 (1.4213)
	S_2	2.65 (1.1334)	4.09 (0.0242)	4.46 (0.0029)
	S_n	3.65 (0.1447)/S_8	5.20 (0.1869)/S_9	6.95 (0.3695)/S_{17}
SO_3_HNN_NH_2_SO_3 (b)	S_1	2.54 (0.0010)	2.97 (1.3510)	3.12 (1.4073)
	S_2	2.65 (1.1574)	4.08 (0.0048)	4.38 (0.0033)
	S_n	3.55 (0.0708)/S_6	5.90 (0.1224)/S_{16}	6.96 (0.4337)/S_{18}
HSO_3_ HNN _NH_2_SO_3 (a)	S_1	2.60 (0.0000)	3.09 (1.3784)	3.21 (1.4477)
	S_2	2.83 (1.1281)	3.98 (0.0009)	4.44 (0.0053)
	S_n	3.28 (0.1963)/S_3	6.68 (0.2263)/S_{20}	7.14 (0.4329)/S_{16}
HSO_3_ HNN _NH_2_SO_3 (b)	S_1	2.65 (0.0018)	3.08 (1.3890)	3.19 (1.4364)
	S_2	2.86 (1.2948)	3.97 (0.0004)	4.35 (0.0037)
	S_n	4.79 (0.0734)/S_{12}	6.55 (0.1368)/S_{18}	7.11 (0.4696)/S_{16}
HSO_3_HNN_NH_2_SO_3H (a)	S_1	2.89 (1.3353)	3.13 (1.4363)	3.26 (1.4763)
	S_2	3.42 (0.0290)	4.32 (0.0224)	4.42 (0.0025)
	S_n	5.26 (0.0746)/S_{14}	6.80 (0.2640)/S_{17}	7.20 (0.3345)/S_{13}
HSO_3_HNN_NH_2_SO_3H (b)	S_1	2.81 (1.2335)	3.05 (1.3286)	3.19 (1.3780)
	S_2	3.38 (0.0201)	4.26 (0.0042)	4.36 (0.0043)
	S_n	4.54 (0.1758)/S_7	5.11 (0.1931)/S_6	7.45 (0.2743)/S_{18}
HSO_3_HNNH_NH_2H_SO_3H (a)	S_1	2.03 (0.0072)	2.43 (0.0408)	2.67 (1.2842)
	S_2	2.36 (0.2236)	2.56 (1.3002)	2.84 (0.0638)
	S_n	2.38 (0.5720)/S_3	6.06 (0.0388)/S_{17}	6.98 (0.1485)/S_{20}
HSO_3_HNNH_NH_2H_SO_3H (b)	S_1	2.03 (0.0085)	2.43 (0.0647)	2.66 (1.2756)
	S_2	2.35 (0.3540)	2.56 (1.2476)	2.85 (0.0614)
	S_n	2.49 (0.4730)/S_4	6.21 (0.0646)/S_{19}	6.49 (0.1404)/S_{16}

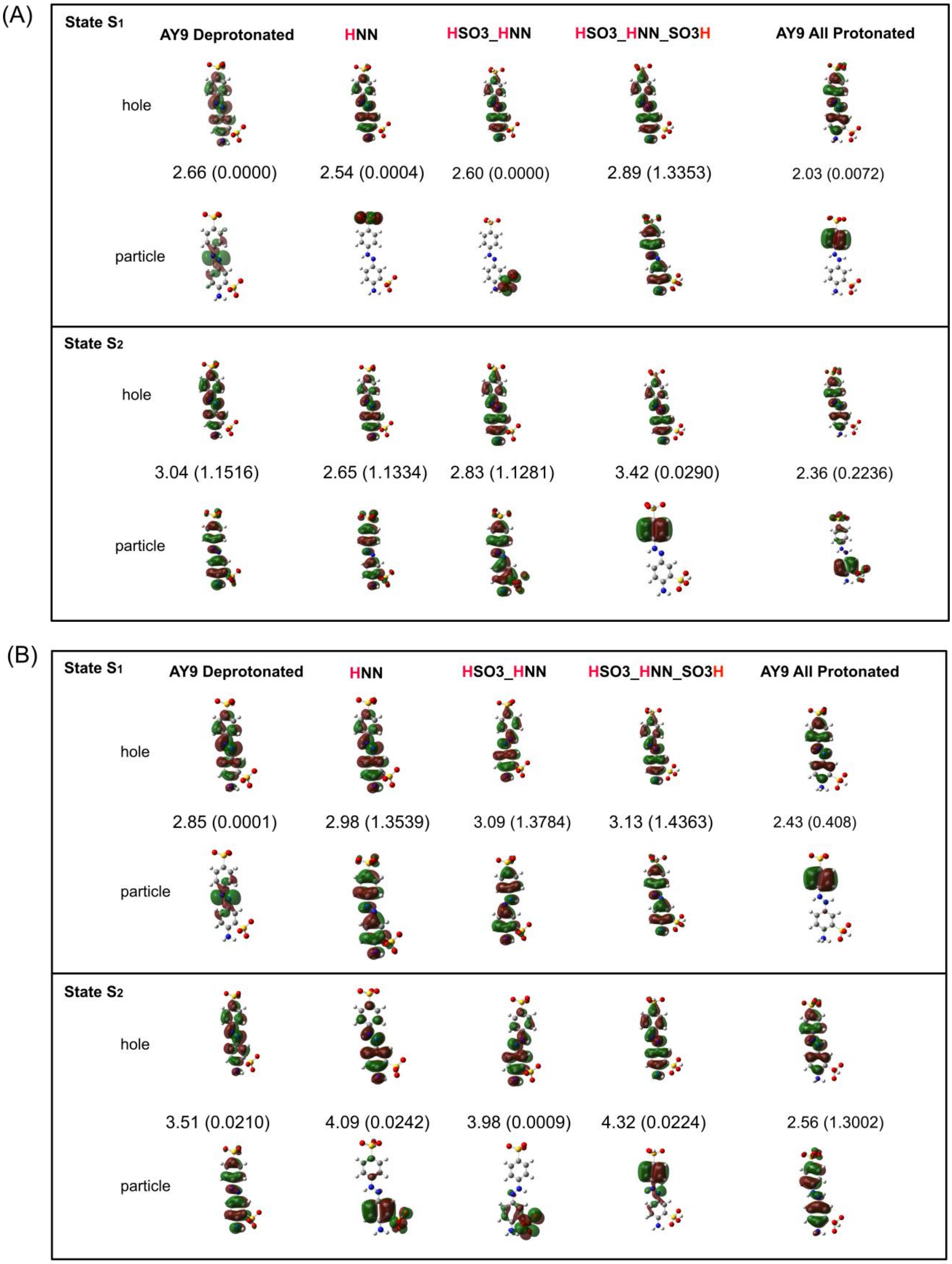

Figure S10 Results of NTO analysis of two excited states S_1 and S_2 for the AY9 molecule of different protonation sites, using a 6-31g(p, d) basis set, CPCM (implicit), and using functionals: (A) B3LYP or (B) CAM-B3LYP.

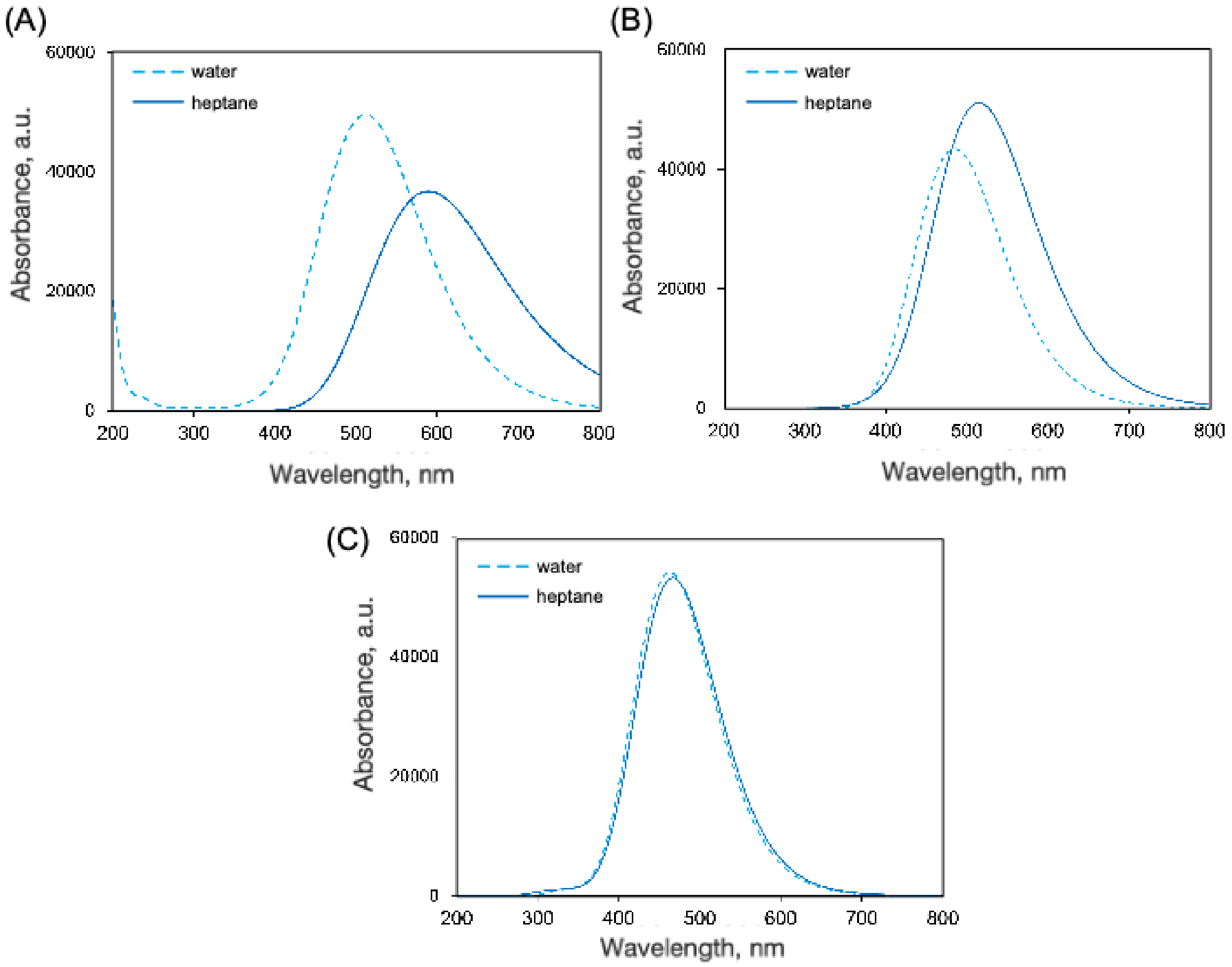

Figure S11 Theoretical predictions of absorption spectra for AY9 in solvents water and heptane, from TD-DFT calculations with a 6-31g(p,d) basis set, model CPCM (implicit), and functionals: (A) B3LYP, (B) CAM-B3LYP, and (C) LC-ωHPBE of full protonation (sites 1–5).

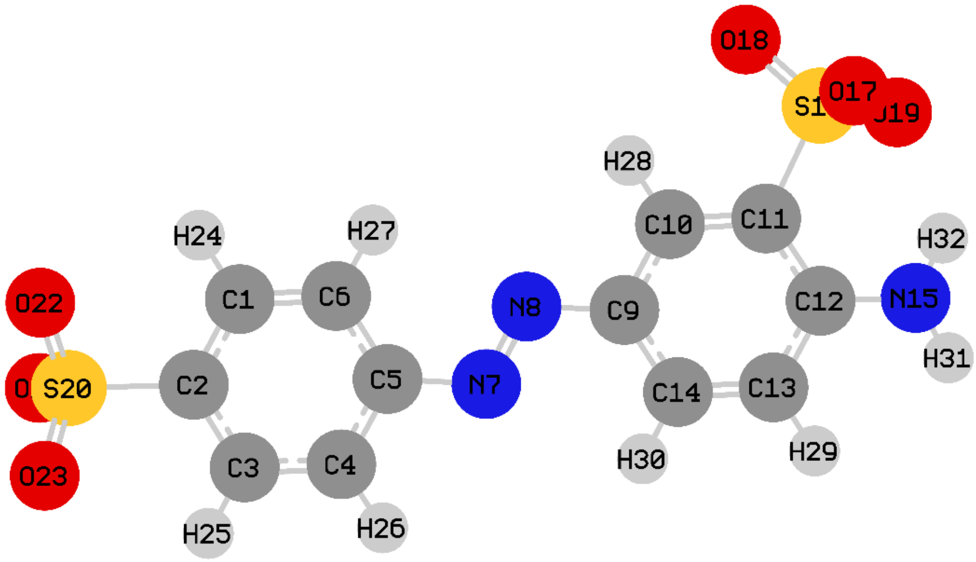

Figure S12 Labels and numbering of atoms in AY9 for DFT calculations.

DFT Analysis: The NBO method was used to analyze bonds and electron density of the AY9 molecule. Nitrogen atoms #7 and #8 (Fugure S13, previous page) of the azo-group in AY9 were analyzed for both fully deprotonated and fully protonated states using B3LYP and 6-31g(d,p). Between N7 and N8 two molecular bonds (BD) are formed with occupancies 1.98 and 1.91, suggesting the bonding character of the orbitals.

The first BD is formed by an overlapping of AOs from N7 and N8, characterized by significant contributions from p-orbitals (69.9% for N7 and 69.8% for N8), and from s-orbitals (29.9% for N7 and 30.1% for N8). Analysis indicated that BD1 is a σ-MO formed by sp2-hypridized AO orbitals of N7 and N8. The second BD is formed by overlapping of AOs that have almost pure p-character (99.7% for N7 and for N8), indicating that BD2 is π-MO formed by p AOs of N7 and N8.

N7 and N8 both have lone pairs (LP) of electrons. For N7 the occupancy is 1.96 indicating that the LP is almost fully occupied, which is typical for a non-bonding MO. LP7 has 38.0% s- and 61.9% p-character, indicating that LP7 most likely resides in a sp2-hypbrid AO. For LP8 the occupancy is 1.95, indicating a non-bonding MO. LP8 has 37.8% s- and 63.1% p-character.

The characteristics of BDs and LPs allow us to expect for azo group to have a planar geometry that is observed in DFT optimized molecule structure. Analogous analysis of NBOs of N15 indicates sp3-hypridization of the nitrogen atom of the amino group for un-protonated AY9, that can be observed as a tetrahedral geometry in the DFT optimized structure. Upon protonation, due to resonance (Figure 6A), the nitrogen of the amino group becomes sp2-hybridized and the group takes on a planar geometry.
